# Non-Cationic Proteins Are Associated with HIV Neutralizing Activity in Genital Secretions of Female Sex Workers

**DOI:** 10.1371/journal.pone.0130404

**Published:** 2015-06-19

**Authors:** Kenzie D. M. Birse, Amy L. Cole, Taha Hirbod, Lyle McKinnon, Terry B. Ball, Garrett R. Westmacott, Joshua Kimani, Frank Plummer, Alexander M. Cole, Adam Burgener, Kristina Broliden

**Affiliations:** 1 Proteomics Unit, National Laboratory for HIV Immunology, JC Wilt Infectious Disease Research Centre, Public Health Agency of Canada, Winnipeg, Manitoba, Canada; 2 Department of Medical Microbiology, University of Manitoba, Winnipeg, Manitoba, Canada; 3 Burnett School of Biomedical Sciences, University of Central Florida College of Medicine, Orlando, Florida, United States of America; 4 Unit of Infectious Diseases, Department of Medicine Solna, Center for Molecular Medicine, Karolinska Institutet, Karolinska University Hospital, Stockholm, Sweden; 5 Centre for the AIDS Programme of Research In South Africa, Doris Duke Medical Research Institute, Nelson R Mandela School of Medicine, University of KwaZulu-Natal, Congella, South Africa; 6 National Microbiology Laboratory, Public Health Agency of Canada, Winnipeg, Manitoba, Canada; 7 Department of Medical Microbiology, Kenyatta National Hospital, University of Nairobi, Nairobi, Kenya; University of Maryland School of Medicine, UNITED STATES

## Abstract

**Objective:**

Cationic proteins found in cervicovaginal secretions (CVS) are known to contribute to the early antiviral immune response against HIV-infection *in vitro*. We here aimed to define additional antiviral factors that are over-expressed in CVS from female sex workers at high risk of infection.

**Methods:**

CVS were collected from Kenyan HIV-seronegative (n = 34) and HIV-seropositive (n = 12) female sex workers, and were compared with those from HIV-seronegative low-risk women (n = 12). The highly exposed seronegative (HESN) sex workers were further divided into those with less (n = 22) or more (n = 12) than three years of documented sex work. Cationic protein-depleted CVS were assessed for HIV-neutralizing activity by a PBMC-based HIV-neutralizing assay, and then characterized by proteomics.

**Results:**

HIV neutralizing activity was detected in all unprocessed CVS, however only CVS from the female sex worker groups maintained its HIV neutralizing activity after cationic protein-depletion. Differentially abundant proteins were identified in the cationic protein-depleted secretions including 26, 42, and 11 in the HESN>3yr, HESN<3yr, and HIV-positive groups, respectively. Gene ontology placed these proteins into functional categories including proteolysis, oxidation-reduction, and epidermal development. The proteins identified in this study include proteins previously associated with the HESN phenotype in other cohorts as well as novel proteins not yet associated with anti-HIV activities.

**Conclusion:**

While cationic proteins appear to contribute to the majority of the intrinsic HIV neutralizing activity in the CVS of low-risk women, a broader range of non-cationic proteins were associated with HIV neutralizing activity in HESN and HIV-positive female sex workers. These results indicate that novel protein factors found in CVS of women with high-risk sexual practices may have inherent antiviral activity, or are involved in other aspects of anti-HIV host defense, and warrant further exploration into their mode of action.

## Introduction

HIV-seronegative women who have had frequent sexual HIV exposures over many years have been shown to mount an efficient innate and HIV-specific immune response in the genital tract mucosa despite their uninfected status [1]. HIV-neutralizing IgA and innate immune factors including cationic proteins, CCR5-ligands and antiproteases have been found in genital tract secretions of various HIV-exposed seronegative (HESN) cohorts such as female sex workers and HIV serodiscordant couples [2–6]. Many cationic proteins have a synergistic antimicrobial effect and HIV neutralizing capacity *in vitro* [7], and constitute an important arm of innate immunity against pathogens at genital mucosal surfaces. Expression of innate immune proteins is normally regulated by various factors including sex hormones, inflammatory conditions and the local microflora [8–12]. Furthermore, their functional capacity can vary depending on their local distribution, concentration and interaction with other molecules at the mucosal site [13]. The mechanisms of immune protection from heterosexual transmission of HIV infection are thus complex and have not been extensively studied *in vivo*. Mucosal samples collected from HESN populations are a useful tool to complement *in vitro* experiments and non-human primate studies to explore such local immune activities.

As genital secretions are known to contain many hundreds, if not thousands, of unique proteins [14,15], many with antimicrobial and antiviral functions, it is likely that multiple factors contribute to the host defense against HIV. This has however not been fully explored since most studies have only examined predefined factors. Understanding the complexity of factors contributing to HIV neutralizing activity can be performed comprehensively using systems biology approaches. In the present study we examined cervicovaginal secretions (CVS) from HIV-seronegative and HIV-seropositive female sex workers as well as CVS from HIV-seronegative low-risk women not participating in sex work. CVS samples were tested for HIV neutralization and were analyzed via mass spectrometry to identify proteins that could potentially be contributing to this effect. Here, this study identified novel protein factors within the cationic protein-depleted fraction of the mucosa that may be involved in the intrinsic antiviral nature of cervicovaginal compartment and host defense against heterosexual HIV transmission in addition to the well-described effects of various cationic proteins [7,16].

## Material and Methods

### Study population

HESN and HIV-seropositive female sex workers were recruited through the Pumwani Sex Worker Cohort [17] and HIV-seronegative low-risk women were recruited through a Maternal Health Clinic based at the Pumwani Maternity Hospital [18]. All sex worker participants enrolled were currently active in sex work whereas all low-risk women enrolled reported no history of sex work. Although their partner was not tested for HIV serology these women were considered to be significantly less exposed to HIV than the study participants in the sex worker groups. All women underwent a full physical examination and sexually transmitted infections (STI) testing at enrolment; diagnostics included HIV serology, urine for *Chlamydia trachomatis* and *Neisseria gonorrhoeae* molecular testing; syphilis serology; and Gram stain for Nugent scoring. All participants were provided with HIV/STI prevention counseling, male and female condoms, family planning services, treatment of STIs, medical care for acute and chronic illnesses, access to adequate diagnostic testing and referral for specialist consultant and/or hospitalization as needed. This study was reviewed and approved by the research ethics boards at Kenyatta National Hospital (Nairobi, Kenya); The Regional Ethical Review Board in Stockholm, Sweden; and the Research Ethics Board of the University of Manitoba (Winnipeg, Canada). All study participants provided written informed consent.

### Cervicovaginal Fluid Collection

CVS were collected by rotating a cotton swab 360° in the outer part of the endocervix and by rotating a different swab across the vaginal wall. Both swabs were placed in the same tube containing 0.5 ml sterile phosphate buffered saline, which was immediately placed on ice. The samples were then transported from the clinic to the laboratory on ice and centrifuged at 800 g for 10 minutes at 4°C. Supernatants were separated from the cell pellet and stored at -80°C.

### HIV neutralization assay

HIV neutralization assays were performed according to a predefined protocol and neutralization cut off [19,20]. Prior to this assay, the IgG fraction of the CVS samples from the HIV seropositive women was removed to avoid IgG-mediated HIV-specific neutralizing activity [21]. The IgG-depleted fraction was stored at -80°C until use. The other study groups were by definition HIV IgG seronegative and their CVS samples were therefore left intact. For the HIV neutralizing assay, an R5 tropic primary isolate of HIV subtype A (isolate 92UG037; AIDS Research and Reference Reagent Program, Division of AIDS, NIAID, NIH) was used. The CVS sample volume did not allow assessment with additional virus isolates. To account for variations in TCID_50_ depending on PBMC donor variability, three viral dilutions were used in each assay and a TCID_50_ of 10–50 was finally selected for evaluation. Duplicate wells of 75 μl of each virus dilution and 75 μl of each sample fraction (undiluted) were incubated for 1 hour at 37°C followed by addition of a mixture of 1 x 10^5^ PHA-P-stimulated PBMC from two donors. After 24 hour incubation at 37°C, the cells were centrifuged; unbound virus was washed away, and 200 μl of fresh medium were added to each well. On day 3, 120 μl of medium was discarded and replaced with new medium, after which day 6 supernatants were collected for analysis of virus production with a p24 antigen ELISA (Vironostika HIV-1 Antigen; Electra-Box Diagnostica AB, Stockholm, Sweden). Neutralization was defined as a ≥67% reduction of p24 antigen in the supernatant as compared with p24 antigen content when the virus isolate was incubated in the presence of a standard pool of Swedish HIV seronegative heat-inactivated plasma samples diluted 1:20.

### Selective depletion of cationic proteins from CVS

Carboxymethyl (CM) weak cation exchange resin (Bio-Rad) was used to deplete cationic proteins from vaginal fluid [7]. The CM resin was pre-equilibrated by washing 6 times with a buffer resembling vaginal fluid in electrolyte composition (60mM NaCl, 20mM potassium phosphate, pH 6) [22]. Equal volumes of CVS from four to six HIV seronegative donors were centrifuged and pooled before the extraction of cationic proteins. From each pool, 0.5 mL CVS was reserved and stored at -80°C as “unprocessed CVS”. The remaining volume from each pool was CM-extracted by mixing with equilibrated CM resin at 4°C overnight in an end-over-end tumbler. Centrifugation (15,000xg, 4°C, 3min) enabled collection of the CM-depleted CVS supernatant, which was then cleared of residual resin by additional centrifugations and stored at -80°C. The CM resin sediment was washed 5 times with 25mM ammonium acetate, pH 7, and then cationic proteins were extracted by incubating the washed resin with 5% acetic acid in a tumbler at 4°C. Extracted proteins were collected and stored at -80°C after 2 h, and the resin was extracted a second time by incubation with more acetic acid overnight in a tumbler at 4°C. The first and second cationic extracts were pooled, clarified of residual resin, concentrated by vacuum centrifugation, and restored to the original volume. All samples were stored at -80°C until use.

### Protein Digestion and Preparation for MS Analysis

The protein content of the cationic protein-depleted CVS samples was measured by standard BCA protein assay (Novagen). Ten micrograms from each sample was added to ammonium bicarbonate buffer (100mM, Sigma) to a total volume of 100μl. Five microliters of dithiothreitol (DTT, 200mM, Sigma) was added to each sample. Samples were vortexed, centrifuged and incubated at 60°C while gently shaking for 1 hour. Twenty microliters of iodoacetamide (200mM, Sigma) was then added to each sample. Samples were vortexed, centrifuged and incubated at room temperature for 30 minutes in the dark. Eleven microliters of DTT (200mM) was added after this 30 minute incubation to neutralize any further iodoacetamide activity (to prevent alkylation of trypsin). Samples were again vortexed, spun down and incubated at room temperature in the dark for 1 hour. Trypsin (Promega) was added (2ug/sample) and incubated at 37°C overnight. Peptides were dried via vacuum centrifugation the following morning and stored at -80°C. The samples were then cleaned of salts and detergents by reversed-phase liquid chromatography (high pH RP, Agilent 1200 series micro-flow pump, Water XBridge column) using a step-function gradient such that all peptides elute into a single fraction for each sample. The fractions were then dried via vacuum centrifugation and kept at -80°C until analyzed by mass spectrometry.

### Mass Spectrometry Analysis

Fractions were re-suspended in 2% acetonitrile (Fisher Scientific), 0.1% formic acid (EMD Canada) and injected into a nano-flow Easy nLC II connected in-line to an LTQ Orbitrap Velos mass spectrometer with a nanoelectrospray ion source at 2.35 kV (Thermo Fisher Scientific, San Jose, CA, USA). The peptide fractions were loaded onto a C_18_-reversed phase trap column (2 cm long, 100 μm inner diameter, 5 μm particles) with 100% buffer A (2% acetonitrile, 0.1% formic acid) at 3 μl/min for a total volume of 30 μl, and then separated on a C_18_-reversed phase column (15 cm long, 75 μm inner diameter, 3 μm particles). Both columns were packed in-house with ReproSil-Pur C_18_-AQ resin (Dr. Maisch) and fritted with Kasil. Peptides were eluted using a linear gradient of 2–32% buffer B (98% acetonitrile, 0.1% formic acid) over 120 min at a constant flow rate of 250 nl/min. Total LC/MS/MS run-time was 160 minutes, including the loading, linear gradient, column wash at 95% buffer B, and the equilibration.

Data were acquired using a data-dependent method, dynamically choosing the top 10 abundant precursor ions from each survey scan for isolation in the LTQ (2.0 *m/z* isolation width) and fragmentation by CID (35% normalized collision energy, with 10 ms activation time). The survey scans were acquired in the Orbitrap over *m/z* 300–1700 with a target resolution of 60000 at *m/z* 400, and the subsequent fragment ion scans were acquired in the LTQ Velos over a dynamic *m/z* range. The lower threshold for selecting a precursor ion for fragmentation was 1000 ions. Dynamic exclusion was enabled using a list size of 500 features, a *m/z* tolerance of 15 ppm, a repeat count of 1, a repeat duration of 30 s, and an exclusion duration of 15 s, with early expiration disabled. Lock Mass was used with polysiloxane. All spectra were processed using Mascot Distiller v2.4.3.1 (Matrix Science) and database searching was done with Mascot v2.4.0 (Matrix Science). Searches were against the SwissProt database (2013–04). The minimum number of unique peptides required for protein identification was set to one peptide and false discovery rates (Prophet) were required to be below 1% for both peptide and protein identification. Relative protein levels were calculated using label-free analysis based on precursor ion peak intensities calculated using Progenesis LC-MS v.4.0 (Nonlinear Dynamics). Complete details regarding the proteomic data set including normalized abundance, number of unique peptides identified and percent coverage are available in S1 Table and are available online (http://figshare.com/articles/Non_cationic_proteins_are_associated_with_HIV_neutralizing_activity_in_genital_secretions_of_female_sex_workers/1428626).

### Statistical & Bio-functional Analysis

Protein abundances were normalized by mean division and log_2_ transformation. Statistical analysis was performed by Student’s T-tests (Perseus, v1.3.0.4, Max Planck Institute of Biochemistry). The significance threshold was set as α = 0.05 and fold changes greater than or equal to 2 (Log_2_ Fold Change = 1). Statistically significant results were entered into Ingenuity Pathway Analysis (Qiagen), Database for Annotation, Visualization and Integrated Discovery (DAVID) v6.7 [23,24] and examined using the UniProt website to determine bio-functional associations. A minimum of two associated proteins and Benjamini-Hochberg corrected Fisher’s Exact Tests were used to determine significant bio-functional associations.

## Results

### Demographic data of the study groups

Analysis of CVS was performed by pooling samples from five to seven women into the following study groups: HIV-seronegative low-risk women (low-risk, n = 12 into 2 pools), HESN female sex workers (n = 34 into 6 pools, divided according to more (2 pools) or less (4 pools) than 3 years of sex work), and HIV-seropositive female sex workers (HIV positive, n = 12 into 2 pools). Sample pooling was required due to the limited quantity of CVS collected and the amounts required for the subsequent assays included in this study. The pools were designed to contain comparable numbers of samples from women in the early and late menstrual cycle stage, and comparable numbers of women using hormonal contraception (oral contraceptives, n = 8, injectable contraceptives, n = 7 or no hormonal contraception use, n = 43) to limit potential confounding effects of the influence of sex hormones on the protein composition (data not shown). None of the samples represented cases of ongoing infections with *Chlamydia trachomatis*, *Neisseria gonorrhoeae* or syphilis. Women with clinical signs of genital ulcers or inflammation were excluded from the study. Bacterial vaginosis (here defined as gram stain for Nugent scoring ≥7) was diagnosed in 0 of 12 low-risk women, in 5 of 24 women in the HESN<3yr sex worker group, in 1 of 12 women in the HESN>3yr sex worker group, and in 1 of 12 women in the HIV-seropositive sex worker group.

### Cationic protein-depleted CVS samples of the FSW study groups can neutralize HIV

**Fig 1:**
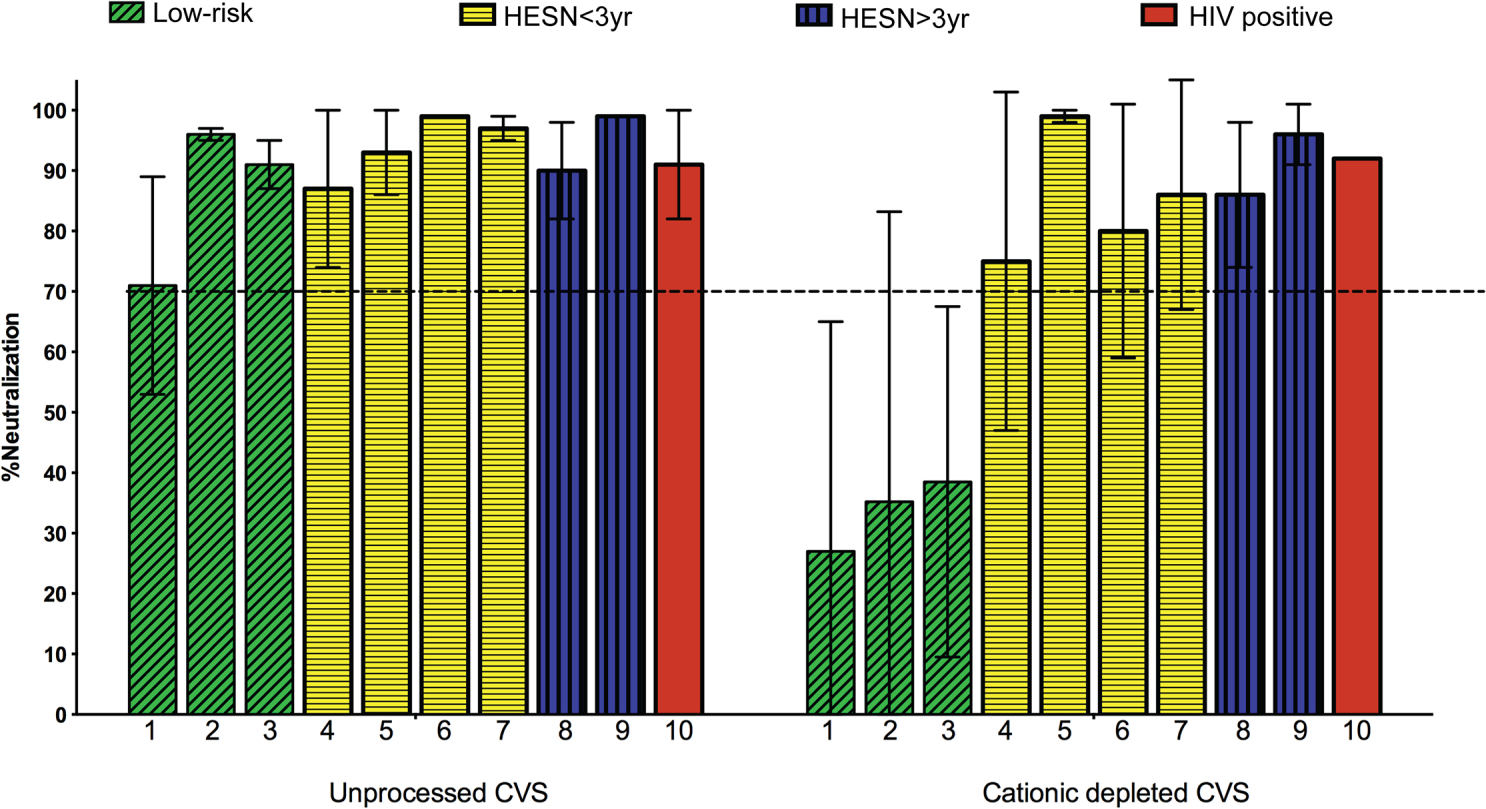
HIV neutralizing activity in unprocessed and cationic protein-depleted cervicovaginal secretions. The HIV neutralizing activity of unprocessed cervicovaginal secretions (CVS) and cationic protein-depleted CVS, respectively, was assessed in pooled samples from the study groups. Green bars: HIV seronegative low-risk women (Low risk) [25]; Yellow bars: HIV seronegative women with less than 3 years of reported active sex work (HESN<3yr); Blue bars: HIV seronegative women with more than 3 years of reported active sex work (HESN>3yr); Red bars: HIV seropositive sex working women (HIV positive). The results shown are from two to three representative experiments using duplicate wells (median values ±SEM).

In the first set of experiments, the intrinsic HIV-neutralizing capacity was assessed in the unprocessed sample pools. In a previous study the pools representing the low-risk study group were shown to neutralize HIV as defined by a PBMC-based assay using a primary HIV isolate of the R5 phenotype/subtype A [25]. Likewise, all pools representing the sex worker (HESN>3yr, HESN<3yr and HIV positive) study groups were here shown to neutralize HIV infection using the same experimental conditions (Fig 1). Next, the cationic proteins were selectively removed from the CVS pools while sparing the concentrations of the remaining proteins and other non-positively charged molecules. The removal of cationic proteins was confirmed by AU-PAGE for all pools (data not shown), thus suggesting that biologically relevant concentrations were depleted. Traces of such peptide fragments could be detected by mass-spectrometry; however the fragments detected may represent inactive remnants of the original proteins (data not shown).

In our previous study we reported a lack of HIV-neutralizing activity in cationic protein-depleted CVS of low-risk women [25]. Thus, the majority of the functional activity was mediated by the cationic protein fraction for these low-risk uninfected women. To test whether this effect was the same in high-risk groups, the sex worker study groups were assessed using the same experimental conditions and showed significant HIV neutralizing activity despite the depletion of the cationic protein fractions (Fig 1). The sample pools representing the HIV positive women had been depleted of IgG prior to the cationic protein-depletion to exclude functional activity mediated by HIV-specific IgG antibodies.

### Proteomic analysis of cationic protein-depleted CVS from all study groups

To define the proteins that were differentially expressed between the study groups, a comprehensive analysis of the depleted fractions was performed by tandem-mass spectrometry proteomic analysis. Relative protein levels were compared using a Student’s *t*-test (α = 0.05, Fold Change≥2). Three comparisons were made: HESN>3yr versus low-risk women (Table 1); HESN<3yr versus low-risk women (Table 2); and HIV positive sex workers versus low-risk women (Table 3), which identified 26, 42, and 11 host differentially abundant proteins, respectively.

**Table 1:** Proteins found to be significantly overabundant (A) and under abundant (B) in the cationic protein-depleted cervicovaginal secretions of HIV-exposed seronegative sex workers (>3 years) compared to HIV-seronegative low-risk controls.

Protein Name	Gene Name	General Function^a^	Log_2_ Fold change difference	P-value^b^
**(A) OVERABUNDANT PROTEINS**				
Cytochrome c *	CYSC	Apoptosis/transport	6.351	0.043
DnaJ homolog subfamily B member 1 *	DNAJB1	Stress response	5.340	0.001
Poly(U)-specific endoribonuclease	ENDOU	Immune response/proteolysis	4.757	0.049
60S ribosomal protein L4	RPL4	Translation	4.311	0.033
Eukaryotic translation initiation factor 2 subunit 2	EIF2S2	Protein biosynthesis	4.085	0.000
Caspase-14	CASP14	Epidermal differentiation	3.445	0.033
Microtubule-associated protein 4	MAP4	Cell division	2.731	0.004
Synaptic vesicle membrane protein VAT-1 homolog	VAT1	Epidermal repair, oxidoreductase activity	2.674	0.027
Tubulin polymerization-promoting protein family member 3	TPPP3	Microtubule bundle formation	2.606	0.017
Aldehyde dehydrogenase, dimeric NADP-preferring	ALDH3A1	Catabolic process	2.415	0.026
SH3 domain-binding glutamic acid-rich-like protein 2	SH3BGRL2	Potential antioxidant defense	2.285	0.046
Nucleobindin-1	NUCB1	Calcium homeostasis	2.283	0.041
Myristoylated alanine-rich C-kinase substrate	MARCKS	Actin Cross-linking	2.191	0.047
Acyl-CoA-binding protein	DBI	Transport	2.146	0.018
Leukocyte elastase inhibitor	SERPINB1	Protease Inhibition	1.871	0.015
UV excision repair protein RAD23 homolog B	RAD23B	Ubiquitin conjugation pathway	1.584	0.010
Vinculin	VCL	Cell adhesion	1.547	0.001
Lysosomal protective protein	CTSA	Proteolysis	1.453	0.032
Thioredoxin	TXN	Innate immune response, oxidoreductase activity	1.330	0.034
Protein S100-A7	S100A7	Innate immune response	1.267	0.025
Barrier-to-autointegration factor *	BANF1	Host-virus Interaction	1.143	0.032
**(B) UNDERABUNDANT PROTEINS**				
Prothrombin	F2	Acute phase response	-4.092	0.014
Protein S100-A11	S100A11	keratinocyte differentiation	-1.915	0.036
Mucin-5AC (Fragments)	MUC5AC	Mucosa component, anti-adhesion	-1.734	0.035
Arachidonate 12-lipoxygenase, 12S-type	ALOX12	Lipid metabolism	-1.373	0.043
Keratin, type II cytoskeletal 6B	KRT6B	Structural	-1.065	0.014

^a^ General functions are based on each protein’s gene ontology obtained from the UniProt website.

^b^ Statistical significance was deduced via Student’s T-test, p < 0.05.

* denotes a known association with HIV-1.

**Table 2:** Proteins found to be significantly overabundant (A) and under abundant (B) in the cationic protein-depleted cervicovaginal secretions of HIV-exposed seronegative sex workers (<3 years) compared to HIV seronegative low-risk controls.

Protein Name	Gene Name		Log_2_ Fold Change Difference	P
**(A) OVERABUNDANT PROTEINS**				
Cytochrome c *	CYCS	Apoptosis/transport	6.344	0.001
DnaJ homolog subfamily B member 1*	DNAJB1	Stress response	5.200	0.012
Hepatoma-derived growth factor	HDGF	Transcription regulation	4.874	0.041
Glutathione S-transferase P	GSTP1	Anti-inflammation	4.446	0.008
Poly(U)-specific endoribonuclease	ENDOU	Immune response/proteolysis	4.097	0.044
60S ribosomal protein L4	RPL4	Translation	4.002	0.014
Quinone oxidoreductase PIG3	TP53I3	Stress response	3.665	0.025
BAG family molecular chaperone regulator 3	BAG3	Anti-apoptosis	3.584	0.009
Serum albumin	ALB	Acute phase response	3.542	0.012
Synaptic vesicle membrane protein VAT-1 homolog	VAT1	Epidermal repair, oxidoreductase activity	3.334	0.001
SH3 domain-binding glutamic acid-rich-like protein 2	SH3BGRL2	Potential antioxidant defense [26]	2.836	0.013
Heterogeneous nuclear ribonucleoprotein Q	SYNCRIP	Host-virus Interaction	2.542	0.006
Myristoylated alanine-rich C-kinase substrate	MARCKS	Actin cross-linking	2.317	0.000
Phosphoglucomutase-2	PGAM1	Glucose metabolism	2.286	0.027
Metallothionein-2 *	MT2A	Ion homeostasis	2.279	0.013
ADP-sugar pyrophosphatase	NUDT5	Nucleotide metabolic process	2.205	0.025
Ubiquitin-40S ribosomal protein S27a *	RPS27A	Innate immune response	2.141	0.004
Nucleobindin-1	NUCB1	Calcium homeostasis	1.971	0.024
STE20-like serine/threonine-protein kinase	SLK	Apoptosis	1.909	0.047
Caspase-14	CASP14	Epidermal differentiation	1.908	0.029
Clathrin light chain B	CLTB	Vesicle-mediated transport	1.849	0.018
Proteasome activator complex subunit 1*	PSME1	Immunoproteasome assembly	1.598	0.023
Lysosomal protective protein	CTSA	Proteolysis	1.564	0.028
Kallikrein-6	KLK6	Proteolysis	1.543	0.018
Barrier-to-autointegration factor *	BANF1	Host-virus Interaction	1.538	0.015
Tubulin polymerization-promoting protein family member 3	TPPP3	Microtubule bundle formation	1.348	0.009
Leukocyte elastase inhibitor	SERPINB1	Protease Inhibition	1.293	0.008
Delta(3,5)-Delta(2,4)-dienoyl-CoA isomerase, mitochondrial	ECH1	Lipid metabolism	1.234	0.004
Cathepsin B	CTSB	Innate immune response/proteolysis	1.198	0.026
Ly6/PLAUR domain-containing protein 3	LYPD3	Cell-matrix adhesion	1.191	0.039
**(B) UNDERABUNDANT PROTEINS**				
Non-histone chromosomal protein HMG-17	HMGN2	Chromatin organization	-6.516	0.024
Coactosin-like protein	COTL1	Defense response to fungus	-5.413	0.017
Keratin, type II cytoskeletal 2 oral	KRT76	Structural	-4.856	0.027
Epidermal growth factor receptor kinase substrate 8-like protein 1	EPS8L1	Cytoskeleton remodeling	-3.318	0.038
Zyxin *	ZYX	Cell adhesion	-2.753	0.025
BPI fold-containing family B member 1	BPIFB1	Innate immune response	-2.388	0.049
Keratin, type II cytoskeletal 2 epidermal	KRT2	Keratinization	-2.188	0.041
Granulins	GRN	Inflammation, embryo implantation	-2.087	0.011
IgGFc-binding protein	FCGBP	Maintenance of mucosal structure	-2.052	0.044
Protein S100-A11	S100A11	keratinocyte differentiation	-1.974	0.003
Mucin-5AC	MUC5AC	Mucosa component, anti-adhesion	-1.708	0.015
Phosphoglycerate mutase 1	PGM2	Glycolysis	-1.028	0.028

^a^ General functions are based on each protein’s gene ontology obtained from the UniProt website.

^b^ Statistical significance was deduced via Student’s T-test, p < 0.05.

* denotes a known association with HIV-1.

**Table 3:** Proteins found to be significantly overabundant (A) and under abundant (B) in the cationic protein-depleted cervicovaginal secretions of HIV-positive sex workers compared to HIV seronegative low-risk controls.

Protein Name	Gene Name	General Function^a^	Log_2_ Fold Change Difference	P-value^b^
**(A) OVERABUNDANT PROTEINS**				
Keratin, type I cytoskeletal 24	KRT24	Structural	5.049	0.015
SH3 domain-binding glutamic acid-rich-like protein 2	SH3BGRL2	Potential antioxidant defense [26]	2.826	0.018
Synaptic vesicle membrane protein VAT-1 homolog	VAT1	Epidermal repair, oxidoreductase activity	2.739	0.036
Myristoylated alanine-rich C-kinase substrate	MARCKS	Actin cross-linking	2.590	0.007
Phosphoglucomutase-2	PGM2	Glucose metabolism	1.735	0.037
60S acidic ribosomal protein P0	RPLP0	Host-virus interaction	1.717	0.039
UV excision repair protein RAD23 homolog B	RAD23B	Ubiquitin conjugation pathway	1.445	0.028
Vinculin	VCL	Cell adhesion	1.060	0.013
**(B) UNDERABUNDANT PROTEINS**				
Keratin, type II cytoskeletal 2 oral	KRT76	Structural	-5.327	0.003
Transcobalamin-1	TCN1	Ion transport	-2.994	0.038
Phosphoglycerate kinase 1	PGK1	Glycolysis	-1.687	0.046

^a^ General functions are based on each protein’s gene ontology obtained from the UniProt website.

^b^ Statistical significance was deduced via Student’s T-test, p < 0.05.

**Fig 2:**
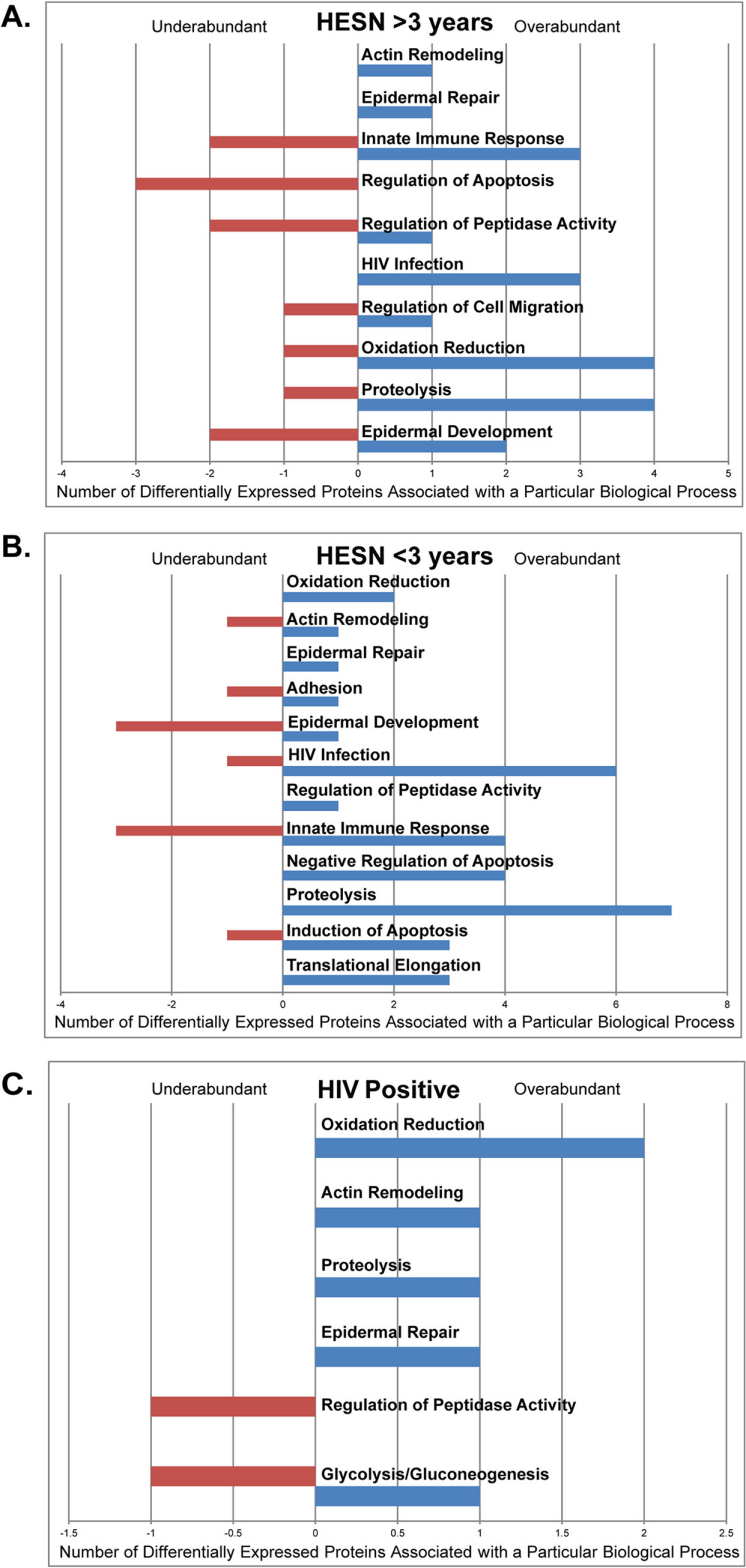
Biological processes of differentially abundant proteins between female sex worker and low-risk groups. **A:** HIV-exposed seronegative female sex workers for a time greater than three years **B:** HIV-exposed seronegative female sex workers for a time less than three years **C:** HIV-seropositive female sex workers (DAVID Bioinformatics Resources 6.7, UniProt) (Blue bars represent overabundant proteins and red bars represent under abundant proteins compared to the low-risk, uninfected control group).

The biological functions associated with the proteins identified as differentially abundant were determined, and there were proteins associated with viral infection and HIV infection in all sex worker groups. Both HESN groups (>3 years and <3 years) were found to differentially express proteins known to have an effect on viral infection (CYCS, DNAJB1, MT2A, RPS27A, CLTB, CTSB, HMGN2, ALB, ZYX) [27–34] with particular proteins known to increase HIV infection according to the results of *in vitro* mRNA silencing experiments (CYCS, DNAJB1, MT2A, ZYX, PSME1, BANF1, RPS27A) [35,36]. Despite the depletion of cationic proteins and the remaining expression of HIV infection enhancing proteins, the CVS of both HESN and HIV-positive groups still maintained a relatively strong capacity to neutralize HIV. Other proteins differentially expressed in these groups compared to the low-risk group included many factors involved in the innate immune response, proteolysis, protease inhibition, oxidation reduction, and others (Fig 2). Several of these proteins have known antimicrobial roles, such as S100 proteins, mucins, leukocyte elastase inhibitor, and thioredoxin. Furthermore protein S100A7, leukocyte elastase inhibitor, and thioredoxin were previously found to be overexpressed in HESN individuals in other proteomic studies [3,15].

Interestingly, three intracellular proteins were found to be commonly overabundant in the cationic protein-depleted secretions of all HIV neutralizing groups: SH3 domain-binding glutamic acid-rich-like protein 2 (SH3BGRL2), Synaptic vesicle membrane protein VAT-1 homolog (VAT-1) and Myristolyated alanine-rich C-kinase substrate protein (MARCKs). All three were found at higher levels in the sex worker groups CVS than the CVS of low-risk women. These proteins are associated with functions such as antioxidant defense, epidermal repair and actin remodeling, respectively. Furthermore, there were two secreted proteins commonly overabundant in the cationic protein-depleted secretions from both HESN groups: Poly(U)-specific endoribonuclease (ENDOU) and Leukocyte elastase inhibitor (SERPIN B1), and two secreted proteins commonly underabundant: Protein S100-A11 (S100A11) and Mucin-5AC (MUC5AC). These factors are of particular interest due to their secreted nature and their known antimicrobial and/or immune-related functions.

## Discussion

This work demonstrates that genital secretions from HIV-seronegative and HIV-seropositive sex workers have an intrinsic capacity to neutralize HIV *in vitro*, which extends previous findings on low-risk uninfected women [7,25,37]. However in contrast, we here showed that when cationic proteins were experimentally depleted from the samples of the female sex workers, the HIV-neutralizing activity remained. Proteomic analysis revealed non-cationic proteins not previously associated with antiviral activity to be present in the HIV-neutralizing secretions. This is the first time any HIV-neutralizing activity has been demonstrated in the cation-depleted protein fraction of cervicovaginal mucosa.

Three proteins were found overabundant and common to all sex worker groups, including SH3BGRL2, VAT-1 and MARCKS. None of these factors have been described to have direct antiviral activity in the current literature, but we here speculate about their potential indirect effects on various pathways involved in the mucosal defense against HIV infection. These proteins are predominantly found intracellularly or within extracellular exosomes. VAT-1 is an integral membrane protein that belongs to a superfamily of medium-chain dehydrogenases/reductases and strongly resembles quinone oxidoreductases and zeta-crystallins [38]. VAT-1 has been shown to be an important calcium-regulated activator of epithelial cells which is required for epidermal repair [39]. Elevated levels of epidermal repair proteins may be important for maintaining an intact physical barrier within the genital tract which is critical for preventing pathogen entry. VAT-1 also has the capacity to function as an oxidoreductase which may further contribute to the overall antioxidant defense in the mucosa of the female genital tract. SH3BGRL2 is believed to be another oxidoreductase belonging to the Thioredoxin-like superfamily as it has a homologous thioredoxin-like fold [26]. Certain Thioredoxin-like family members such as peroxiredoxins have been shown to inhibit HIV replication *in vitro* [40]. Based on SH3BGRL2’s homology with proteins known to affect pathways important for HIV replication and known HIV inhibitors, it is an excellent biomarker for future studies. MARCKS, on the other hand, has functions in cross-linking actin, binding calcium-calmodulin, as well as integrins [41,42]. It has been speculated that these molecular functions contribute to biological processes such as phagocytosis, secretion and/or membrane recycling which are all important defense mechanisms against invading pathogens. As these proteins were all commonly overabundant in the secretions of sex workers compared to low-risk controls, they present as novel biomarkers for further investigations on their direct or indirect role in HIV infection.

Certain proteins that were found to be overabundant in the CVS of the HESN>3yr group have previously been associated with protective immunity in comparable HIV-exposed seronegative cohorts including protein S100A7, leukocyte elastase inhibitor, and thioredoxin [3]. The family of S100 proteins, such as protein S100A7, includes calcium ion-binding proteins that are known to be associated with native antimicrobial activity [43], are derived from epithelial cells, and are secreted in response to external pathogens. Protein S100A7, also known as Psoriasin has been specifically implicated in the innate immunity of the epidermis by enhancing barrier function via the promotion of keratinocyte differentiation and the strengthening of tight junctions [44]. If and when protein S100A7 is induced under inflammatory conditions, it is also known to play a role in the chemotaxis of immune cells including neutrophils and T lymphocytes [45]. This protein’s association with increased barrier integrity could help prevent infection and the recruitment of immune cells may help in viral clearance. However, the fine interplay between the recruitment of HIV target cells for viral clearance versus infection propagation is still not fully understood.

Thioredoxin (TXN) is a disulfide oxidoreductase with innate immune function as mentioned above. TXN plays key roles in reducing reactive-oxygen species-mediated inflammation [46] and promoting cell growth and proliferation [47]. TXN also exhibits dose-dependent chemotactic effects such that at low concentrations it is chemotactic for various immune cells including neutrophils and at high concentrations it suppresses leukocyte chemotaxis and extravasation [46,48]. Furthermore, there is evidence that TXN has the capacity to neutralize HIV in macrophages *in vitro*, however it is noteworthy to mention that the same study found that TXN’s cleavage product, Eosinophil cytotoxicity-enhancing factor had enhancing effects on HIV infection and TXN is often quickly cleaved upon entering circulation [49]. It is therefore, hypothesized that it is more likely that secreted TXN exerts its protective effects by suppressing leukocyte extravasation [46].

Leukocyte elastase inhibitor (SERPINB1), a protein associated with the HESN phenotype in previous literature was also found commonly overabundant in both HESN groups in this study [3]. SERPINB1 belongs to the serine protease inhibitor family of antiproteases, and inhibits both elastase-like and chymotrypsin-like proteases, including Cathepsin G. It has been implicated in providing defense against microorganisms at mucosal surfaces by protecting against protease-mediated inflammatory damage of the epithelium induced during bacterial infections, and promoting wound healing [50]. Other serine protease inhibitors such as Serpin A1, Secreted leukocyte protease inhibitor and Elafin have been associated with anti-HIV functionality *in vitro* [51–54]. Therefore, SERPINB1 may represent a novel target for further study of its potential to neutralize HIV, thus preventing infection.

Poly(U)-specific endoribonuclease (ENDOU) was found commonly overabundant amongst the CVS of HESN individuals within this study. ENDOU is a secreted endoribonuclease that cleaves single-stranded RNA [55]. Based on the current literature, it is mostly commonly expressed in placental tissue and in tumours. Not much is known about this protein making it a novel protein for future studies.

Interestingly, there were also two proteins found commonly under abundant in the CVS of HESN individuals compared to the CVS of low-risk women, protein S100A11 and Mucin 5-AC. S100A11 is also a member of the S100 family, a calcium ion-binding protein expressed by epithelial cells. S100A11 has a dual functionality in keratinocyte growth regulation such that when it is found intracellularly, it suppresses growth and facilitates differentiation and cornification of keratinocytes [56], whereas when it is found extracellularly, it in fact promotes keratinocyte growth [57]. Mucin-5AC (MUC5AC) is a glycoprotein secreted on mucosal surfaces generating another physical protective barrier against microorganisms. MUC5AC has been shown to be induced from epithelial cells by neutrophil elastase in the airways, and may be induced in a similar fashion at other mucosal sites such as the female genital tract [58].

The up-regulation and down-regulation of specific factors at the mucosa of the female genital tract of HIV-exposed sex workers such as those identified in this study may be a result of frequent sexual encounters, their higher risk of genital exposure to seminal fluid from different individuals, and/or from previous STIs. These are all known risk factors for inflammation, disrupted genital microflora and altered composition of the innate immune proteome [59]. It is possible that the up-regulation of some of these factors may be the result of increased Toll-like receptor (TLR) expression in the cervical epithelial cells of HIV-resistant women [60]. Further fractionation is needed to define which of these factors constitute the majority of the non-cationic protein neutralizing activity. Furthermore, only Clade A isolates were tested in this study, therefore we can only attest to the enhanced neutralizing activity of the cationic protein-depleted secretions of female sex workers against these isolates. It is also possible that HIV neutralizing IgA antibodies [19,61] as well as other peptides, below the detection limit of the mass spectrometry analysis, were present and associated with HIV-neutralizing activity. In any case the factors identified in this study and their HIV inhibitory capacity warrant further investigation *in vitro*.

Understanding factors that modulate HIV infection at the female genital tract mucosa is important for the development and implementation of novel antiviral compounds including microbicides. This study increases our knowledge of the neutralizing capacity of genital secretions and identifies new potential antimicrobial factors important for female genital tract immunity.

## Supporting Information

S1 Table Complete details on the proteomic data set. (XLSX)
