# Correction: Non-Cationic Proteins Are Associated with HIV Neutralizing Activity in Genital Secretions of Female Sex Workers

**DOI:** 10.1371/journal.pone.0134196

**Published:** 2015-07-21

**Authors:** Kenzie D. M. Birse, Amy L. Cole, Taha Hirbod, Lyle McKinnon, Terry B. Ball, Garrett R. Westmacott, Joshua Kimani, Frank Plummer, Alexander M. Cole, Adam Burgener, Kristina Broliden

There are errors in the author affiliations. The affiliations should appear as shown here:

Kenzie D. M. Birse^1,2^, Amy L. Cole^3^, Taha Hirbod^4^, Lyle McKinnon^5^, Terry B. Ball^1,2,7^, Garrett R. Westmacott^6^, Joshua Kimani^7^, Frank Plummer^1,2,6,7^, Alexander M. Cole^3^, Adam Burgener^1,2,4^, Kristina Broliden^4^

1 National Laboratory for HIV Immunology, JC Wilt Infectious Disease Research Centre, Public Health Agency of Canada, Winnipeg, Manitoba, Canada, 2 Department of Medical Microbiology, University of Manitoba, Winnipeg, Manitoba, Canada, 3 Burnett School of Biomedical Sciences, University of Central Florida College of Medicine, Orlando, Florida, United States of America, 4 Unit of Infectious Diseases, Department of Medicine Solna, Center for Molecular Medicine, Karolinska Institutet, Karolinska University Hospital, Stockholm, Sweden, 5 Centre for the AIDS Programme of Research In South Africa, Doris Duke Medical Research Institute, Nelson R Mandela School of Medicine, University of KwaZulu-Natal, Congella, South Africa, 6 National Microbiology Laboratory, Public Health Agency of Canada, Winnipeg, Manitoba, Canada, 7 Department of Medical Microbiology, Kenyatta National Hospital, University of Nairobi, Nairobi, Kenya
